# Evaluation of Wetting Behaviors of Liquid Sodium on Transition Metals: An Experimental and Molecular Dynamics Simulation Study

**DOI:** 10.3390/ma17030691

**Published:** 2024-02-01

**Authors:** Na Liang, Xiaogang Fu, Jinquan Zhang, Zhangshun Ruan, Bo Qin, Tengfei Ma, Bin Long

**Affiliations:** China Institute of Atomic Energy, Beijing 102413, Chinazjqciae@163.com (J.Z.); 13811250420@139.com (B.Q.);

**Keywords:** wettability, sodium, transition metals, intermetallic compounds, molecular dynamics simulation

## Abstract

In sodium-cooled fast reactors, the wettability of sodium with materials is closely related to sodium-related operations and the detection accuracy of instruments and meters, so how to achieve the selection of materials with different wettability requirements is a key problem in engineering design. To meet these requirements, the wetting behaviors of liquid sodium with nine transition metals were investigated using scanning electron microscopy (SEM), X-ray diffraction (XRD), and molecular dynamics (MD) simulations. The results show that metals such as zinc and gold, which react with sodium to form intermetallic compounds at the interface, exhibit superior wettability. Followed by the metals that have strong interatomic interactions even though they do not react with sodium or dissolve each other, such as cobalt, nickel and copper, while the wettability of these systems tends to be poor at low temperatures. Systems that do not react with each other or have strong interatomic affinities proved to be the most difficult to wet. Notably, metals with the closest-packed crystal structures of fcc and hcp generally have better wettability than those with a bcc structure. They can be a valuable guide for experimental research and technical control.

## 1. Introduction

The Sodium-cooled Fast Reactor (SFR) system features a fast spectrum reactor and closed fuel recycle system [1]. It is widely recognized that SFRs have a high potential for near-term commercial deployment within the next two decades [2,3,4,5]. As liquid sodium is the reactor coolant of the SFR, the successful operation of the SFR heavily relies on its wettability characteristics. For example, in a sodium loop, it has been found that until full wetting has taken place, sodium will not penetrate crevices and some physical instruments such as flow meters, electromagnetic pumps, and resistor meters will not work satisfactorily. This brings great challenges to the reactor operations and data detection [6,7,8]. Furthermore, in the ultrasonic imaging system of the online detection device in liquid sodium, poor wettability will reduce the acoustic and mechanical coupling between the medium and the detector surface, which directly affects the clarity of the imaging [9,10,11,12,13]. Therefore, excellent wettability is required for such service conditions. However, operations involving filling, draining, charging and some operating tools require a non-wetting behavior even at high temperatures to avoid the adhesion of excessive liquid metal to the surface of the device, leading to removal difficulties and wastage of liquid metal. Therefore, in practical scientific and industrial applications, both extreme wetting and non-wetting surfaces are what is required, thus, achieving the regulation of wettability of liquid sodium is of great importance.

The wetting behavior of liquid sodium has been extensively studied by various research groups. C. C. Addison et al. found that electropolished zinc surfaces showed a critical wetting temperature, whereas abraded surfaces do not, due to the presence of different surface films. They also investigated the role of oxide films in the wetting of iron, cobalt and nickel and suggested that the reduction of oxides by sodium is responsible for promoting wetting [6,14]. This conclusion was further supported by experiments in which surface films such as chromium oxide (Cr_2_O_3_), metal oxides (MnO_2_), or tungsten oxide (WO_3_) were reduced by sodium to the corresponding metals. In addition, they modified the wettability of sodium by adding barium and calcium to modify surface tension [15,16]. Furthermore, D. O. Jordan et al. [17] evaluated non-wetting phenomena based on the degree of miscibility at the solid–liquid interface and the stability of oxide layers on metal surfaces. They proposed the atomic radius ratio as an indicator of the degree of misfit at the atomic scale and then evaluated the wettability of sodium. In addition, M. Kawaguchi et al. [18,19] plated SUS304 stainless steel samples with palladium, nickel, indium and gold, and observed an increase in spreading rate with increasing solubility. They defined a constant, α, representing the spreading rate for each plating material, which was proportional to the logarithm of its solubility. Furthermore, there are also numerous studies of wetting spreading processes and interfacial behavior of other liquid metals using Molecular Dynamics Simulations that capture the wetting properties at the atomic scale [20,21,22,23,24,25].

Despite the many great achievements, few studies have been carried out by systematically studying the wettability of Na with pure metals for the purpose of predicting and screening the wettability of materials with Na. In other words, there is still no clear selection criteria or general assessment strategies for sodium wetting on different materials that have been established. Furthermore, it is crucial to identify the underlying mechanisms and processes at the atomic scale. Herein, the wetting behaviors of liquid sodium with nine transition metals were extensively investigated by measuring contact angles with different times and temperatures. Subsequently, post-experiment microstructural characterization and molecular dynamics (MD) simulations were performed to elucidate the wetting process and the underlying mechanisms, respectively. The ultimate objective of this research was to establish a general strategy for assessing the wettability of liquid sodium, which can be achieved through systematic experimentation and analysis of the wetting process. The results of this investigation can serve as a valuable reference for material screening in practical applications and provide a general strategy for evaluating the wettability of sodium with different materials.

## 2. Experimental and Simulation Details

### 2.1. Materials and Methods

**Figure 1:**
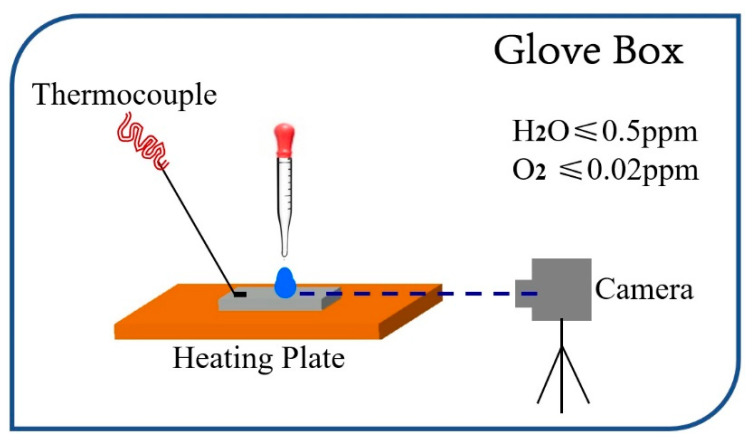
Schematic of the test.

As shown in Figure 1, the experimental device contains a camera, a heating plate, a thermocouple to monitor surface temperature, with all of these in a glove box which provides an inert test environment with low oxygen and low water content. The temperatures used in the experiments were set at 150 °C, 200 °C, 250 °C, 300 °C and 350 °C, respectively. The size of each sample was 30 mm in length, 30 mm in width, and 3 mm in height. The specimens tested included high-purity (99.99%) Cr, Fe, Co, Ni, Cu, Zn, Mo and W, and 304 stainless steel specimens with gold plating. The surface of each specimen, excluding the gold-plated ones, was carefully polished with sandpaper to a roughness of less than 3 μm. The samples were then ultrasonically cleaned with acetone for 30 min and immediately transferred to the glove box for further use. Once the sample surface temperature reached setting and stabilized sufficiently, a 4 mL drop of liquid sodium was dropped centrally onto the surface using a preheated rubber-tipped dropper. Simultaneously, a video camera was activated to record the spreading behavior of the sodium, paying attention to the changes in contact angle over time. Wetting tests lasted 5 h.

After testing, the Na droplet was removed from the surface, and the surface wetting area was observed. The sample was then immersed in ethanol for cleaning purposes and subjected to scanning electron microscopy (SEM, Zeiss-Supra55, Jena, Germany) and X-ray diffraction (XRD, Bruker D8 Advance, Karlsruhe, Germany) analysis to measure the compositions of the surface area.

### 2.2. Simulation Model and Methodology

Although many wetting experiments have been performed, followed by surface micro-analysis, a method to understand the mechanisms and processes of wetting at the atomic scale is still needed. In the present work, molecular dynamics (MD) simulations were employed to investigate the wettability of Na droplets on nine transition metals based on LAMMPS [26] (Large-scale Atomic/Molecular Massively Parallel Simulator) software (LAMMPS 64-Bit 28Mar2023-MPI) in the NVT ensemble. The Nose–Hoover thermostat was employed to control the temperature and pressure [27,28]. The Modified Embedded Atom Method (MEAM) potential was used to describe the atomic interaction for the Na–Cu and Na–Ni systems [29]. For the other seven systems, the Lennard–Jones potential was used for the foreign atoms (Na–Cr [30,31], Na–Fe [32], Na–Zn [30,31], Na–Au [32,33], Na–W [31,32], Na–Mo [31,32], Na–Co [30,32]), while the EAM potential was used for Na–Na [34], W–W [35], Fe–Fe [36], Au–Au [37] and the MEAM potential for Zn–Zn [38], Mo–Mo [39], Cr–Cr [40], and Co–Co [41], respectively. The timestep of MD simulation was Δt = 1 fs. The MD method was applied to all the simulations of Na droplet wetting on different surfaces.

**Figure 2:**
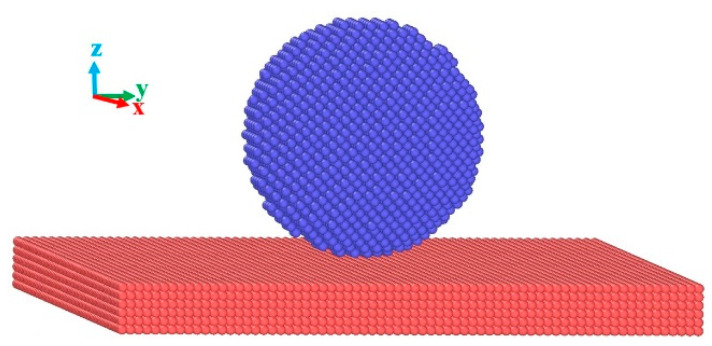
The initial wetting model of the Na droplet on transition metals.

The wetting models of Na droplets on metal substrates were constructed with a distance of 3 Å between them (Figure 2). The transition metal substrates were generated in the bottom of a box with dimensions of 200 × 200 × 200 Å, and the metal crystal of Na was prepared spherically with radius R = 40 Å. The number of atoms was 6826 for the Na droplet, 53,900 for the Fe substrate, 55,445 for the Cu substrate, 59,200 for the Co substrate, 39,204 for the Au substrate, 53,205 for the Cr substrate, 134,460 for the Zn substrate, 58,482 for the Ni substrate, 40,960 for the Mo substrate, and 40,325 for the W substrate, respectively. Nine initial wetting models were constructed as shown in Figure 2. All wetting simulations were run for a total time of 0.5 ns with the NVT ensemble at T = 523 K controlled by the Nose–Hoover thermostat. For each simulation, a time step of 1 fs was set, and periodic boundary conditions were applied in the x, y, z directions. During the simulation, it was found that the temperature output of the droplet and the substrates were different from the set one, which caused the failure of the temperature control. To solve this problem, the temperature of the droplet and the substrate were modified separately, and the actual temperature was finally the same as the setting one.

## 3. Results

### 3.1. Contact Angle Measurement and Surface Composition Analysis

**Figure 3:**
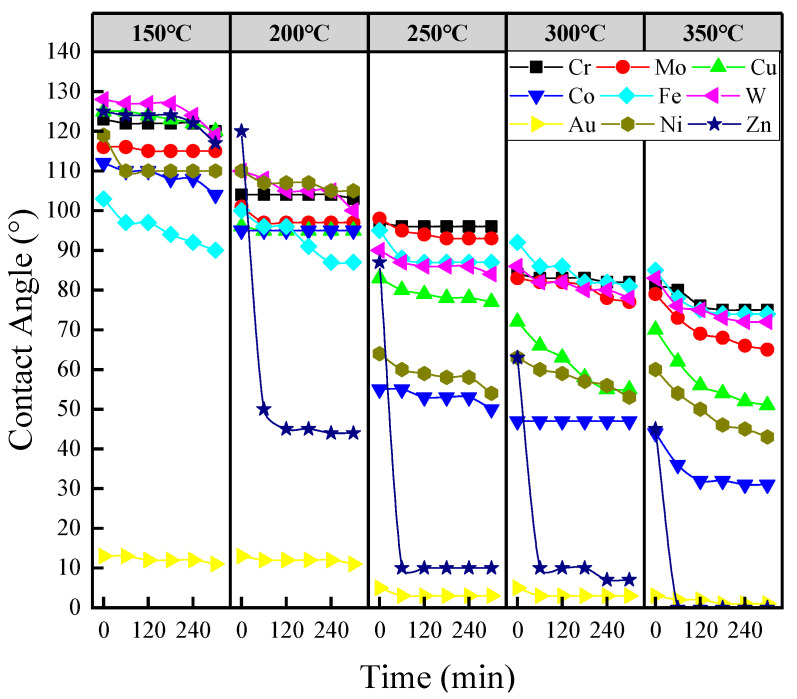
Contact angle versus time curves at 150 °C, 200 °C, 250 °C, 300 °C and 350 °C, respectively.

The wetting behavior of liquid sodium on transition metals, as depicted in Figure 3, was investigated in terms of the variation of the contact angle with time and temperature. According to the definition, “non-wetting” means that, θ > 90°, “wetting” that θ < 90°. It is clearly observed that the contact angles of sodium droplets on the surfaces of all samples decreased with increasing temperature, while notable differences in wettability between the different systems could still be observed. Firstly, the wettability of sodium on Au was consistently superior to other metals, exhibiting immediate wetting at all tested temperatures. This was followed by Zn, non-wetting at 150 °C, and transitioning to wetting after brief time intervals at temperatures above 200 °C, and taking 7 min to reach wetting at 200 °C, 3 min at 250 °C, and almost immediate wetting at 300 °C and 350 °C. Ni, Co and Cu ranked just behind Au and Zn in terms of wettability. All of them showed non-wetting behavior below 200 °C, but a sharp decrease as the temperature increased to 250 °C and above. Finally, the contact angle curves for the remaining four metals were generally between 115° and 127° at 150 °C, 87° and 110° at 200 °C, 84° and 100° at 250 °C, 77° and 95° at 300 °C, and 65° and 85° at 350 °C, respectively. Although the curves appear to be close, there are still distinct trends in the variation of contact angles on different metal substrates. It is noteworthy that the contact angles of Cr and W showed a relatively uniform decrease as the temperature increased. Conversely, no significant decrease in contact angle was observed for Fe within the temperature range tested. Furthermore, Mo showed the highest wettability among the four metals above 350 °C.

**Figure 4:**
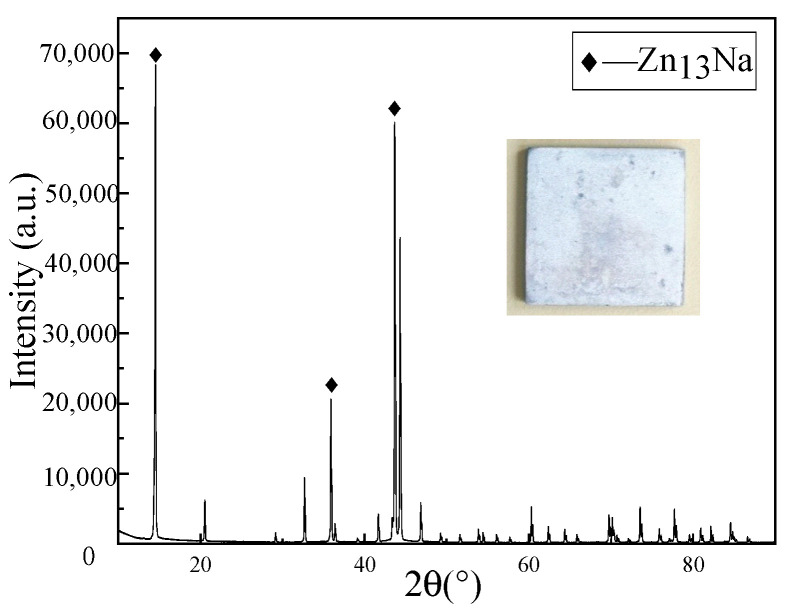
The XRD spectra of the surface of Zn after testing.

**Figure 5:**
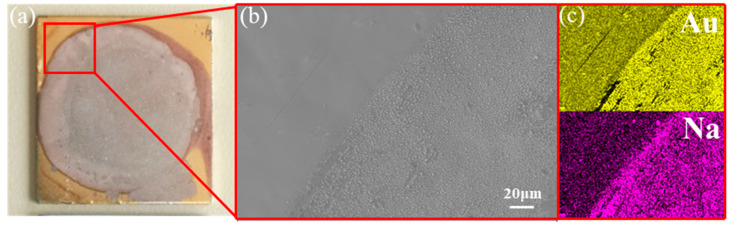
Spreading edge morphology of the Au samples after wetting: (**b**) is the magnified images of the area marked as red rectangles in (**a**), and (**c**) are the elemental distributions of Au and Na in (**b**), respectively.

The surface compositions of the nine transition metals were analyzed after the experiments were completed, as shown by the XRD (Figure 4) and SEM (Figure 5), a layer of the intermetallic compound Zn_13_Na was tested on the surface of Zn, and elements of Na and Au were also detected on the gold-plated substrate, confirming that intermetallic compounds were formed between the two systems, while none were found in the other Na–Metal systems. Comparison of the phase diagrams further confirmed that the only Zn_13_Na formed is between the Na–Zn system, while a series of intermetallic compounds, AuNa_2_, AuNa, Au_2_Na and Au_5_Na are formed in the Na–Au system, which is fully consistent with the experimental results. This suggests that there is a strong relationship between the intermetallic and wettability. The role of the intermetallic in wetting in metallic systems has been studied by Naidich [42], Eustathopoulos [43] and Protsenko et al. [44]. They have two views on this study, some considered that the reaction energy liberated at the interface can strongly improve the driving force of wetting, while others believed that the formation of the intermetallic at the interface has a beneficial but rather limited effect on wetting. While from the results of our experiments, it can be concluded that for the Na–metal system, the reaction between them to form intermetallic compounds plays a crucial role in the wettability, and this may be applicable to other alkali metals as well.

Different Na–Metal systems exhibit different wetting behaviors, which in turn reflect different physicochemical processes at the interfaces. For Zn and Au, although both exhibited superior wettability, the spreading process of sodium droplets on them still showed a distinct difference. As shown in Figure 3, the wetting of Na on Zn would undergo a process, while the wetting of sodium on Au occurred almost instantaneously. The difference in wetting behavior between Zn and Au results from the different interfacial reaction processes and the different physical and chemical nature of the metals. For the Zn–Na system, when the temperature reaches above 97.8 °C, Zn_13_Na is the only intermetallic compound formed [45], which means that Zn_13_Na would be formed at the moment of Na droplet contact with Zn, but due to the relatively active nature of Zn, a thin film of zinc oxide would be formed quickly on the surface after grinding and polishing treatment in air. Thus, at the initial stage of contact, it is a process of the Na droplet reacting with the zinc oxide film and reducing Zn continuously, and only when enough Zn is reduced with a content higher than 92%(at), would Zn_13_Na be formed. The time at which wetting occurrs depends on when enough Zn has been reduced. In contrast, the nature of Au is stable enough to avoid oxidation, which means that a Na droplet would contact Au directly without the process of reduction of its oxide film. In addition, a series of intermetallic compounds exist between the Na–Au systems, and Au_2_Na can be formed at room temperature [46]; thereby, reaction will occur at the moment of contact, resulting in less time-dependence. Therefore, it can be concluded that it is the driving force of the interfacial reaction that induced the rapid wetting.

**Figure 6:**
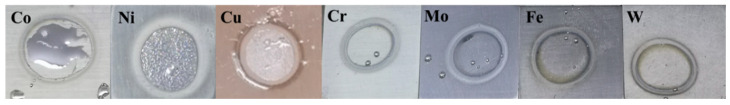
The sample surfaces after removal of Na droplets after 5 h of wetting.

The wettability of Co, Ni and Cu is second only to that of Au and Zn. It can be observed that after the Na droplets were removed after testing, a uniform liquid film of sodium appeared on the surface of all three groups of metals covering the test area, indicating that the liquid sodium formed a relatively tight bond with the solid metal interface (Figure 6). In contrast, after the Na droplets were removed from Fe, Cr, Mo and W, no uniform film of liquid Na remained on the surfaces, implying that Na droplets did not form a tight fit with these samples. For these non-reactive metal systems, what dominates the difference in wettability? It is difficult to trace the atomic-scale wetting process and determine its microscopic spreading state only in an experimental way. Therefore, molecular dynamics simulations were performed.

### 3.2. Spreading Behaviors of Na Droplets on Substrates

**Figure 7:**
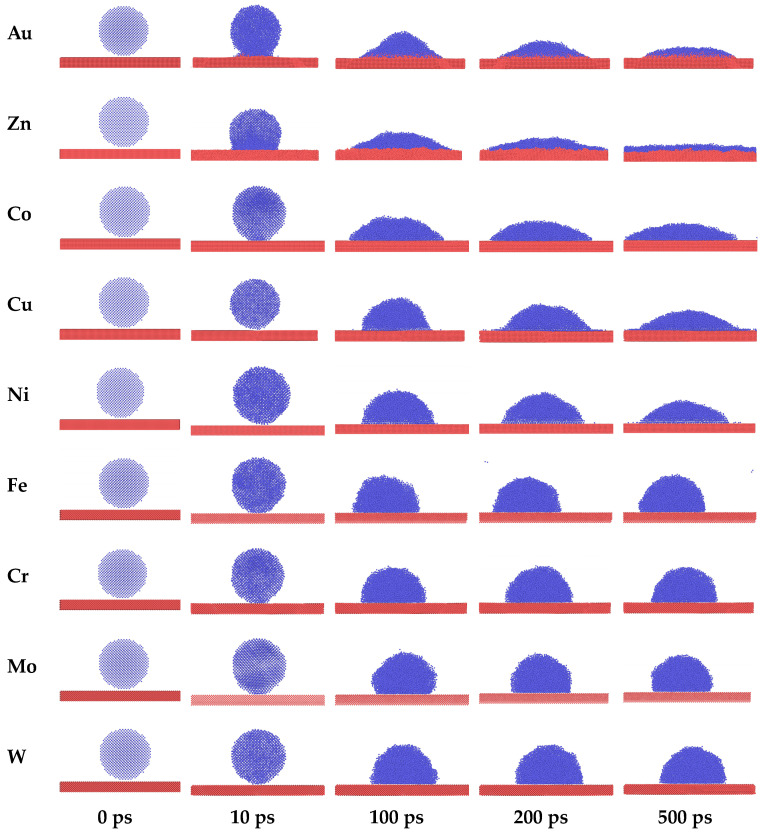
Side views of cross-section snapshots of Na droplets on nine surfaces at t = 0 ps, t = 100 ps, t = 200 ps, and t = 500 ps, respectively.

**Figure 8:**
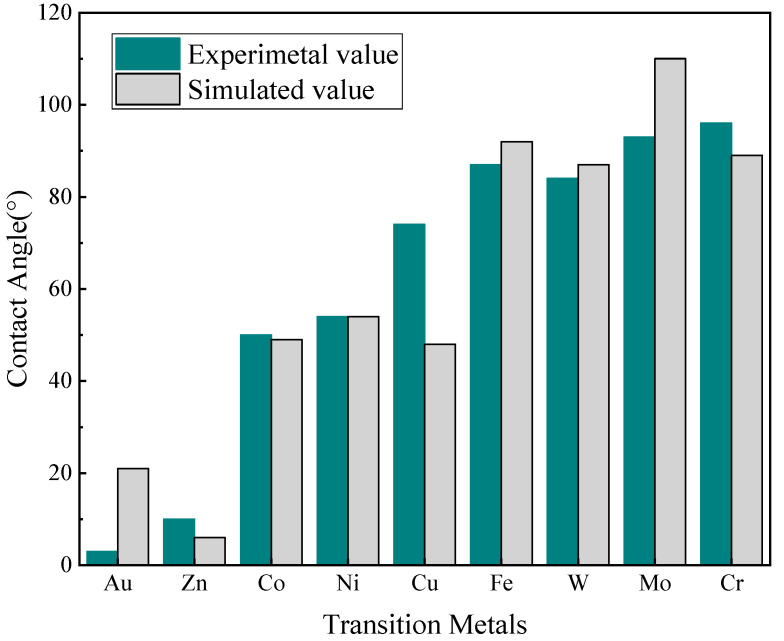
Bar diagram of final contact angles: experimental values versus simulated ones.

The snapshots of the spreading behavior of Na–metal (Na–M) systems at 250 °C are shown in Figure 7. Taking the initial structure after equilibrium as the wetting starting point, the contact angles of all systems gradually decreased with the simulation time until the systems equilibrated and no longer changed. The contact angles of Na droplets on Au and Zn changed dramatically ever 100 ps, and atomic exchange, i.e., interfacial alloying, occurred at the solid–liquid interface. The contact angles of Na droplets on Co, Ni, and Cu changed relatively slowly, while the spreading state of Na droplets on the Fe, Mo, W and Cr were close to equilibrium after 100 ps, with almost no more change thereafter. As shown in Figure 8, the simulated and experimental values of the final state contact angle are in good agreement, indicating that the atomic trajectories during the simulation can be used to describe the spreading process of the actual Na droplets.

**Figure 9:**
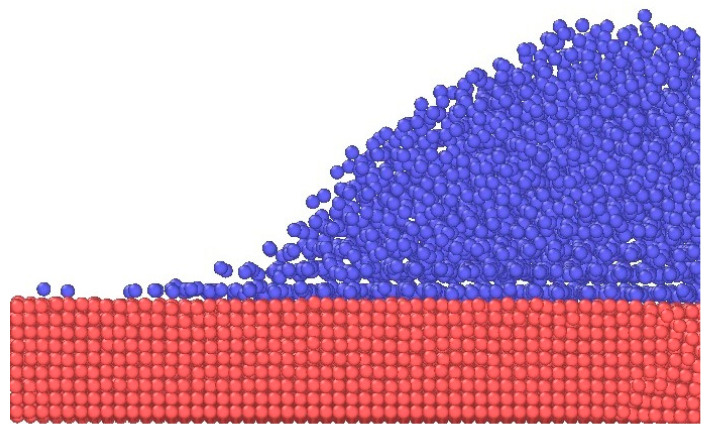
The side view after spreading for 250 ps of a Na droplet on Cu at 250 °C.

**Figure 10:**
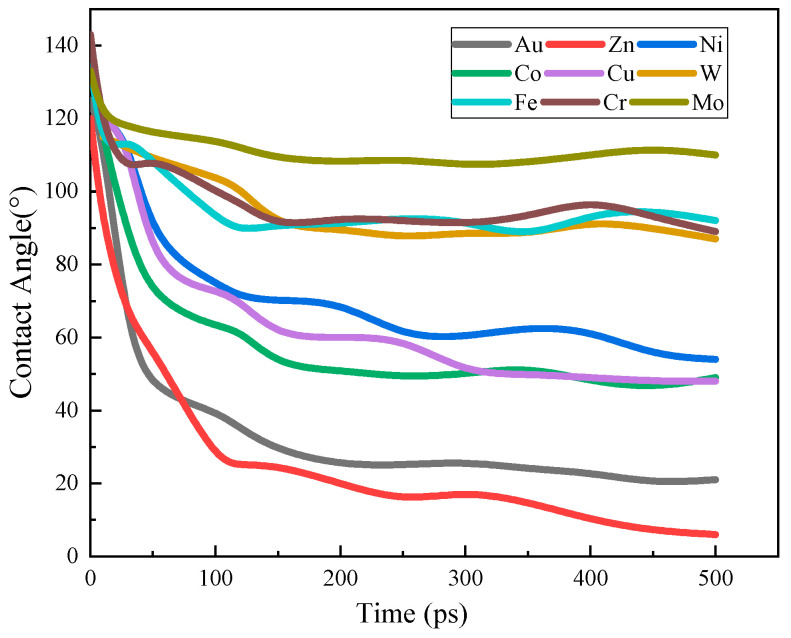
Dynamic contact angles for Na droplets on nine metals.

During the wetting process, it was observed that unlike the way sodium spreads on other metals, a single sodium atomic precursor film was found on the Cu surface after 250 ps (Figure 9). Subsequently, the Na atoms at the top of the Na droplets continue to spread forward on the precursor film causing the contact angle to decrease remarkably at this stage (Figure 10). This suggests that the appearance of the precursor film influences the spreading behavior of the Na droplet and is an indication of better wettability.

### 3.3. End-State Wetting of Na Droplets and Interfacial Atomic Behavior

Under ideal wetting conditions, the precursor film in metal–metal systems is more likely to be in the form of an adsorbed film. It has been suggested that the spreading of the precursor film is based on the diffusion of the first molecular layer (or a thicker molecular layer) on the solid, which occurs between non-reactive systems. It shows that the formation of the Na precursor film on Cu is complemented by its good wettability.

**Figure 11:**
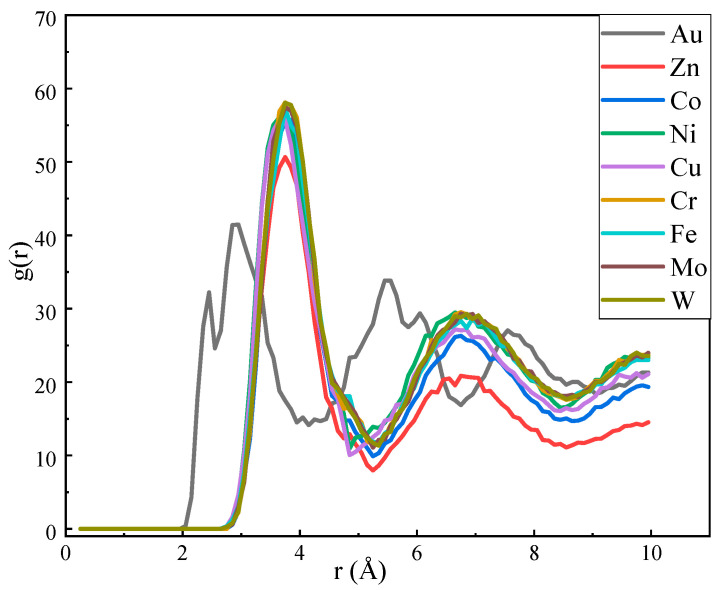
Interatomic Na–Na RDF on the nine metal surfaces.

The phenomenon of whether a precursor film appears or not when the Na droplets spread on the nine metals indicates that the spreading details of the Na atoms on the nine metals are different, and to further understand the structure and atomic order information of the Na droplets, the Na–Na radial distribution function (RDF) of the final state sodium atoms on the substrates was analyzed. As shown in Figure 11, except for the Au–Na system, all the other systems have a prominent peak at the distance of 3.75 Å, indicating the first dense atomic layer, while the value is 3 Å on the Au substrate, indicating that the sodium atoms on the Au substrate are more tightly arranged and the interatomic interactions among the Na atoms are stronger compared to the other systems. In contrast, the distribution of sodium atoms in other systems is more loosely arranged.

**Figure 12:**
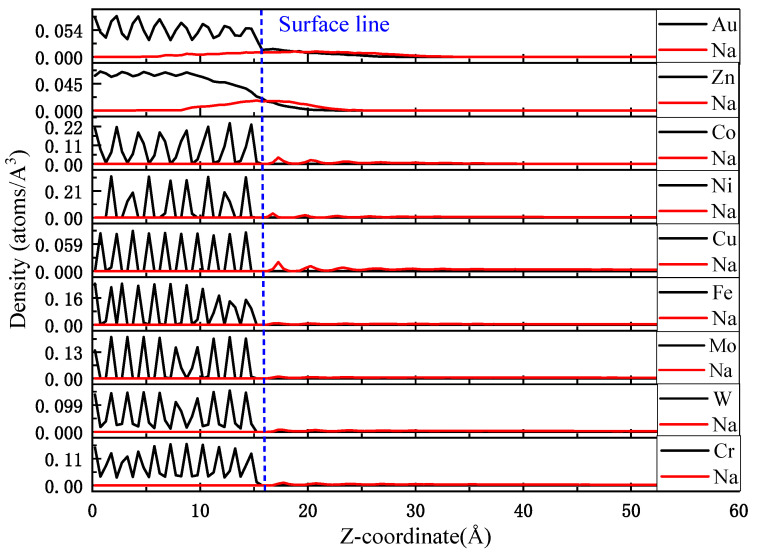
The density distribution profiles along the z axis for Na droplets on nine surfaces at 250 °C.

For a further understanding of the distribution of atoms at the solid–liquid interface, the substrate and Na droplets were layered along the z-axis direction, with each layer having a thickness of 0.5 Å, and the number densities of each layer statistically calculated. As shown in Figure 12, for both Na–Au and Na–Zn systems, the black and red lines have undergone interfacial exchange, indicating that interdiffusion of substrate atoms and Na atoms occurs at the interface, which suggests that the high degree of interatomic bonding of atoms between the Na–Au and Na–Zn systems is demonstrated from a computational point of view, providing the possibility of generating intermetallic compounds in terms of bonding distances. No interfacial exchange of solid–liquid atoms was observed between any of the other systems. The first peaks of the Na droplets on Co, Ni and Cu substrates are high compared to those on Fe, Mo, W and Cr, indicating that the number of Na atoms near the surface of the substrates is larger, i.e., the number of spreading Na atoms is large and the wettability performance excellent. The peak heights of the sodium droplets on Fe, Mo, W and Cr are all lower, implying that the Na droplets are poorly dispersed and have weaker wettability.

**Figure 13:**
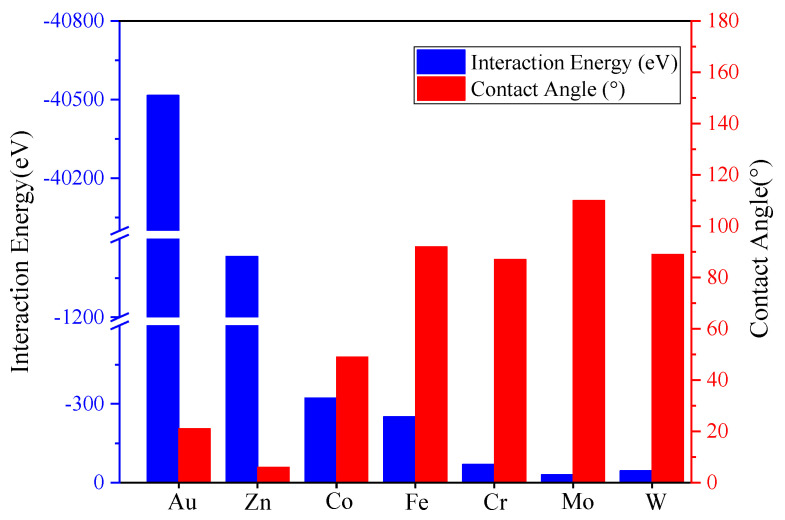
Bar diagram of interaction energies versus contact angles.

For the Na–M systems, regardless of whether the Na droplets are interfacially alloyed or merely adsorbed on the surface, it is the interaction energy between the solid–liquid atomic groups that drives the spreading process and ultimately determines the spreading extent of the liquid metal. The interaction energy is calculated as ΔE = E_total_ − (E_Na_ + E_Metal_), where ΔE is the interaction energy of the solid–liquid atomic group, and E_total_, E_Na_, and E_Metal_ represent the energy parameters of the system, the Na, and the metal, respectively. The more significant the negative value of the interaction energy, the lower the energy in the system from the interaction and the system is more likely to be stabilized, i.e., the system has a stronger tendency to react. Since the potential parameter type used for Cu–Na and Ni–Na is the MEAM potential, which is not supported by the MD algorithm for calculating interaction energies between groups of atoms, only the interaction energies for the other seven systems were calculated. The values are listed in Table 1, which shows that the Na–Au system has the negative maximum value of −40,515 eV, which is far higher than the other systems, followed by the Na–Zn system with a value of 1432 eV. The relationship between interaction energy and wettability is shown in Figure 13, where the contact angle increases and the wettability decreases as the interaction energy decreases. Therefore, it can be concluded that the atomic interaction of the solid–liquid atoms dominate the occurrence of wetting, whereas the contact angle, as an indicator of the atomic interaction, directly reflects its value.

In reviewing the metals that have different wettability with Na, it appears that certain intrinsic properties of the materials are somehow related to wettability. For example, the crystal structure. To summarize the results of the experiments and simulations, it is found that crystals with the most compact structures of fcc and hcp, which have densities of 0.74 and coordination number of 12, generally have better wettability than the bcc metal, which has a density of 0.68 and a coordination number of 8. The phenomena indicate that, for the solid metal itself, the tightness of the internal atomic bonding and the compactness of the crystal structure both play a positive role in wetting, which provides a preliminary judgement of materials with different wettability. It also provides a new idea for controlling wettability by microstructural modification of materials.

## 4. Discussion

In this study, we focused on the influencing factors that lead to differences in wettability behaviors, and presented a general strategy for selecting such materials with sodium which can be used as a guide for material screening. According to the above discussion, it can be summarized as follows:

Optimal wettability occurs in systems that are capable of forming intermetallic compounds by interfacial reactions, where the reaction provides the driving force that can drive rapid wetting. Therefore, in conjunction with the phase diagram, the wettability of a system can be judged by whether intermetallic compounds can be formed between the systems or even by the temperature at which the reaction occurs. Followed by the metals that do not react with Na to form intermetallic compounds, but have strong interactions between them, it is noteworthy that the wettability tests all performed poorly at low temperatures in these systems. The poorest are those that do not react with Na or interact weakly with it.

Based on the laws presented in the experimental and computational data, it can be concluded that the fundamental driving force that dominates the occurrence of wetting is the interaction energy between the systems, and that this ultimately determines the degree of wetting that can be achieved. On the other hand, the contact angle is an indicator of the interaction between solid–liquid heterogeneous atoms, which can accurately measure the value of the interaction energy in a macro-scale way. From this point of view, it appears that the compactness of the crystal structure may be a material property that relates whether it is susceptible to producing large interactions with other materials. The generalized strategy derived from this study may have some positive implications for solving the similar wettability problems of other alkali metals and even other liquid metals.

## Figures and Tables

**Table 1:** Interaction energy between the Na droplet and transition metals.

System	Na–Cr	Na–Fe	Na–Co	Na–Zn	Na–Mo	Na–W	Na–Au
Interaction Energy (eV)	−69	−251	−322	−1432	−42.63	−45	−40,515

## Data Availability

Data are contained within the article.
